# Integrated single‐cell transcriptomics reveals miR‐941 targets CLDN4 to inhibit malignant progression of thyroid cancer through the PI3K/AKT signaling pathway

**DOI:** 10.1002/ccs3.70123

**Published:** 2026-10-27

**Authors:** Jinghua Zhou, Tiancheng Fu, Guoxiang Huang, Guangfeng Luo, Chengcheng Wang, Yanze Wang, Ziyang Ding, Xiarong Hu, Yudong Li, Zhen Li

**Affiliations:** ^1^ Department of Thyroid Surgery The Tenth Affiliated Hospital Southern Medical University (Dongguan People's Hospital) Dongguan Guangdong China; ^2^ Northern Jiangsu People's Hospital Affiliated to Yangzhou University Jiangsu China; ^3^ The Fourth General Surgery Department of the Fourth Affiliated Hospital of China Medical University Shenyang China

**Keywords:** CLDN4, miR‐941, PI3K/AKT pathway, single‐cell transcriptomics, thyroid cancer

## Abstract

Claudin‐4 (CLDN4), a key tight junction protein, has been implicated in tumor progression; however, its functional role and regulatory mechanisms in thyroid cancer remain unclear. This study aimed to investigate the contribution of CLDN4 to malignant progression and its interaction with the tumor–immune microenvironment. Single‐cell and bulk transcriptomic data were integrated to identify CLDN4‐associated malignant epithelial subpopulations. CLDN4 expression was validated in 25 paired papillary thyroid carcinoma (PTC) tissues by qRT‐PCR, western blotting, and immunohistochemistry. Functional assays were performed using CLDN4‐overexpressing and CLDN4‐knockdown B‐CPAP and TPC‐1 cells. The miR‐941–CLDN4 interaction was verified by luciferase reporter assays. PI3K/AKT signaling and macrophage polarization were evaluated in vitro, and tumor growth was assessed in a xenograft mouse model. A CLDN4‐high epithelial subpopulation was identified and associated with advanced tumor stage and PI3K/AKT activation. CLDN4 was upregulated in PTC tissues, whereas miR‐941 was downregulated. High CLDN4 expression correlated with larger tumor size, lymphatic metastasis, and vascular migration. CLDN4 overexpression promoted proliferation, migration, colony formation, and apoptosis resistance, whereas knockdown produced the opposite effects. miR‐941 directly targeted CLDN4 and attenuated its oncogenic effects. CLDN4 activated PI3K/AKT signaling, while PI3K inhibition reversed these phenotypes. CLDN4 also promoted M2 macrophage polarization and suppressed M1 polarization. In vivo, CLDN4 enhanced tumor growth, which was inhibited by PI3K blockade. miR‐941 suppresses CLDN4 to inhibit PTC progression through modulation of the PI3K/AKT pathway. These findings provide mechanistic insights into tumor progression and immune microenvironment remodeling, highlighting CLDN4 as a potential therapeutic target.

## Graphical abstract

The combined single‐cell and functional analysis depicts that miR‐941 targets CLDN4 and activates the regulatory axis that drives the malignancy of thyroid cancer through the PI3K/AKT pathway.

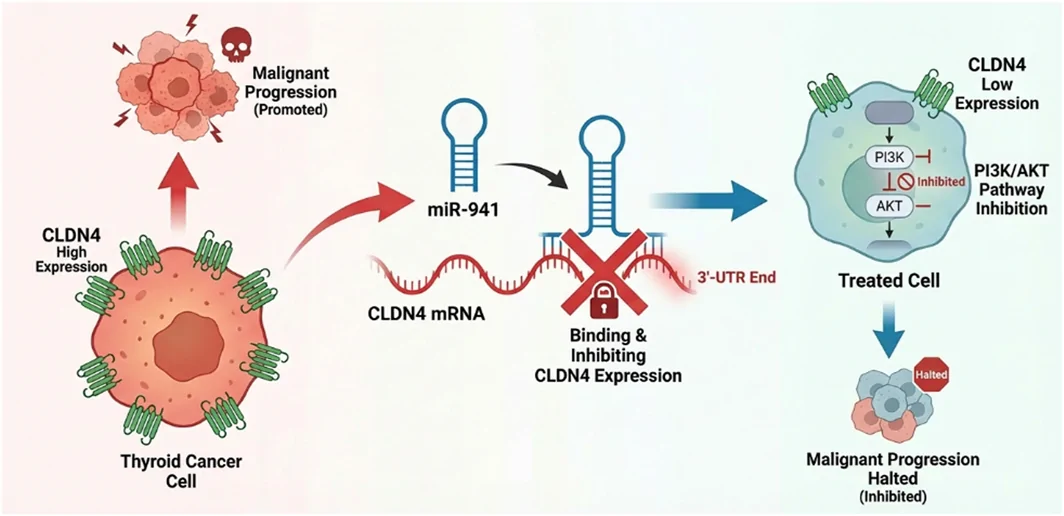

## 1 INTRODUCTION

Thyroid cancer ranks among the world's most rapidly increasing cancers, and the number of women suffering from it is approximately threefold that of men.^1^ In 2020, approximately 586 000 new cases were reported in the world, which made it the 10th most common cancer in the world.^2^ Most thyroid cancers come from follicular epithelial cells. Papillary thyroid carcinoma (PTC) is the most common type, accounting for about 80% of all cases, while follicular carcinoma and anaplastic carcinoma account for 10%–15% and 1%–2%, respectively.^3^, ^4^ Although surgery, radiotherapy, and chemotherapy can often successfully treat the initial disease, the results are usually not optimistic for those who are seriously ill or do not respond to treatment, such as those with anaplastic or medullary carcinoma.^5^ These malignant tumors are important causes of thyroid cancer death and also bring great economic and social problems. Therefore, it is an urgent problem to find out the biological mechanism behind them and find more effective treatment methods.

There are 24 genes named claudin (CLDN) in the human genome, which encode 26 proteins with four transmembrane regions (20–27 kDa) that are very similar in structure. These proteins are the basic materials that constitute the important functional unit of tight junctions (TJs).^6^, ^7^ Recent data show that CLDN4 plays multiple roles in tumor development, depending on the specific context. Elevated levels of CLDN4 promote epithelial‐mesenchymal transition (EMT) and cancer spread in prostate cancer,^8^ breast cancer,^9^ and ovarian cancer,^10^ which is related to poor outcomes. Conversely, in gastric cancer ^11^ and pancreatic ductal adenocarcinoma,^12^ high expression of CLDN4 will inhibit tumor invasion and be associated with better prognosis. CLDN4 is located on the outer surface of the unconnected basement membrane of cancer cells, making it a feasible target for targeted therapy and promoting the development of antibody therapy against this protein.^13^ ASP1002 is a new bispecific antibody that simultaneously binds CLDN4 and 4‐1BB. Now, it is in the first phase of a clinical trial (NCT05719558; Table 2), targeting patients with solid tumors that express CLDN4.^14^ Although some independent studies have found that CLDN‐1, ‐4, and ‐7 are altered in various thyroid malignancies,^15^ whether CLDN‐4 is a suitable therapeutic target for thyroid cancer remains to be determined.

The human‐specific microRNA, miR‐941, stands out for its substantial expression in the prefrontal cortex and cerebellum, where it plays a pivotal role in regulating gene expression in our species' cognitive center.^16^ Functionally speaking, this particular microRNA appears to be connected to both the Hedgehog and insulin signaling pathways, suggesting it may have had a hand in steering human evolution toward greater longevity.^16^ What's more, miR‐941 shows up in spades during human embryonic development and is also found at elevated concentrations across various cancer cell lines, most notably those derived from liver, lung, breast, and prostate tumors.^17^, ^18^, ^19^, ^20^ Notably, its expression is downregulated during stem cell differentiation and is regulated by DNA methylation. Exposure to the DNA methyltransferase inhibitor 5‐aza‐2′‐deoxycytidine increases its expression, thereby inhibiting the proliferation, migration, and invasion of liver cancer, gastric cancer, and colorectal cancer cells.^17^, ^18^, ^19^, ^20^ MiRNA‐941 may affect the metabolic function, inflammatory response, cell growth, and other biological mechanisms of people with acute coronary syndrome by controlling the elements related to insulin signal transduction, MAPK pathway, T cell receptor activation, and related processes.^20^ Therefore, miR‐941 may represent a promising diagnostic biomarker and therapeutic target for a variety of highly expressive tumors. Knockdown of SREBP1 in various thyroid tumor cell line subtypes results in a significant upregulation of miR‐941 expression,^21^ but the biological effect of miR‐941 on thyroid tumor cells has not been clarified.

Through comprehensive analysis, our study provided multiple lines of evidence supporting a pro‐tumorigenic role of CLDN4 in thyroid cancer. We also confirmed that miR‐941 can directly bind and inhibit CLDN4, thus promoting tumor development by activating the PI3K/AKT signaling pathway. These findings enhance our understanding of the molecular mechanism behind the development of thyroid cancer and provide a solid scientific and preclinical basis for the therapeutic strategies against CLDN4.

## 2 MATERIALS AND METHODS

### 2.1 Patients and tissue samples

Thyroid cancer tumors and adjacent normal tissues were collected from patients who underwent surgical treatment at the Tenth Hospital Affiliated to Southern Medical University. Fresh‐frozen tissues from 25 patients were immediately frozen in liquid nitrogen and stored at −80°C, and were used for qRT‐PCR, western blotting, and immunohistochemical (IHC) staining. In addition, paraffin‐embedded tissue sections and matched clinicopathological data were collected from 82 patients with thyroid cancer and were used for the analysis of the correlation between CLDN4 expression and clinicopathological characteristics. All samples were obtained through surgery, and the diagnosis of thyroid cancer was based on clinical and pathological results. The research protocol was approved by the Research Ethics Committee of the Tenth Affiliated Hospital of Southern Medical University (KYKT2025‐110). Prior to study commencement, written informed consent was obtained from each participant.

### 2.2 Single‐cell RNA sequencing data analysis

Single‐cell RNA sequencing (scRNA‐seq) data of PTC were obtained from the GEO database, including four paired tumor and adjacent normal samples from GSE184362 and two paired samples from GSE250521, yielding a total of six patients and twelve samples (Table 1). Data processing and analysis were performed using the Seurat package in R. Cells with fewer than 200 detected genes, more than 7500 genes, or mitochondrial gene content greater than 10% were excluded. Genes expressed in fewer than three cells were removed. After quality control, data were normalized using the LogNormalize method, and highly variable genes were identified. Samples from different patients and datasets were integrated using canonical correlation analysis to correct for batch effects. Dimensionality reduction was performed by principal component analysis followed by UMAP, and unsupervised clustering was conducted using a graph‐based algorithm. Cell types were manually annotated based on canonical marker genes. Epithelial cells were further extracted for subclustering and differential expression analysis between tumor cells and normal epithelial cells using the Wilcoxon rank‐sum test. Functional enrichment analysis was performed using KEGG pathway analysis, and cell–cell communication between tumor cells and immune subsets was analyzed using established ligand–receptor interaction frameworks.

**TABLE 1:** Sample information.

Sample name	GEO	Group
PTC3_T	GSE184362	Tumor
PTC3_P	GSE184362	Normal
PTC5_T	GSE184362	Tumor
PTC5_P	GSE184362	Normal
PTC8_T	GSE184362	Tumor
PTC8_P	GSE184362	Normal
PTC9_T	GSE184362	Tumor
PTC9_P	GSE184362	Normal
PTC3	GSE250521	Tumor
N3	GSE250521	Normal
PTC4	GSE250521	Tumor
N4	GSE250521	Normal

### 2.3 TCGA bulk RNA‐seq data acquisition and integrative analysis

Bulk RNA‐seq expression profiles and corresponding clinical information of thyroid carcinoma were obtained from The Cancer Genome Atlas (TCGA‐THCA) database. To reduce inter‐individual variability and technical bias, only patients with paired primary tumor and matched normal thyroid tissues were included for downstream analysis. Raw count data were normalized, and differential expression analysis between tumor and normal tissues was performed using a standard bulk RNA‐seq differential analysis pipeline. Genes with statistically significant differential expression were identified after multiple testing correction.

To integrate bulk and single‐cell transcriptomic data, differentially expressed genes identified from TCGA bulk RNA‐seq analysis were intersected with epithelial cell–specific genes derived from the scRNA‐seq dataset. The overlapping gene set was subsequently used for downstream functional analyses to identify key epithelial‐related genes associated with PTC progression.

Gene Expression Profiling Interactive Analysis (GEPIA) database analysis: CLDN4 expression was analyzed using the GEPIA database (http://gepia.cancer‐pku.cn/). A total of 512 thyroid tumor samples and 337 normal thyroid tissues were included in the analysis. The expression of CLDN4 in tumor vs. normal tissues was compared using the GEPIA online tool with default settings. Statistical significance was determined by the GEPIA built‐in analysis, with *p* < 0.05 considered statistically significant.

### 2.4 Cell lines and cell culture

We used these thyroid cell lines, and the specific information is in Table 2. These five different cells were put into T‐75 culture bottles (Corning, Oneonta, NY, USA), and each bottle contained 3 million cells. Then we put them in a warm and humid environment containing 5% carbon dioxide and kept the temperature at 37°C. These cells were cultured in either RPMI‐1640 or DMEM medium (Cytiva, Marlborough, MA, USA), supplemented with 10% fetal bovine serum (FBS; Cytiva) and 1% penicillin‐streptomycin (Thermo Fisher Scientific, Waltham, MA, USA).

**TABLE 2:** Information of Cell lines.

	Nthy‐ori 3‐1	TPC‐1	B‐CPAP	MDA‐T85	K1
Purchased from	Procell	Immocell	Procell	ATCC	FuHeng
Product code	CL‐0817	IM‐H153	CL‐0575	CRL‐3354	FH1196
Culture conditions	RPMI‐1640 + 10%FBS	DMEM + 10%FBS	RPMI‐1640 + 10%FBS	DMEM + 10%FBS	DMEM + 10%FBS
Species	Homo sapiens	Homo sapiens	Homo sapiens	Homo sapiens	Homo sapiens
Sex	Female	Female	Female	Female	Female
Tissue of origin	Normal thyroid tissue	Papillary thyroid carcinoma	Papillary thyroid carcinoma	Papillary thyroid carcinoma	Papillary thyroid carcinoma
Purchased date	2024.6.1	2023.9.21	2023.9.21	2024.6.18	2024.6.18
RRID	CVCL_2659	CVCL_6298	CVCL_0153	MDA‐T85	CVCL_2537

We confirm that the cell line was authenticated for the described experiments and has not been previously reported as misidentified or contaminated, and the cell line was free of *mycoplasma* contamination for the described experiments.

### 2.5 Cell transfection

Lentiviruses for CLDN4 overexpression (CLDN4‐OE, NM_001305.5) and CLDN4 silencing were designed and constructed by General Biol (Anhui, China). For CLDN4 knockdown, three independent short hairpin RNA (shRNA) sequences targeting different regions of the CLDN4 transcript (shCLDN4‐#1, shCLDN4‐#2, and shCLDN4‐#3) were designed, together with a non‐targeting scrambled shRNA serving as the negative control (KD‐NC). The shRNA primers used were listed in Table 3. B‐CPAP were infected with the lentiviruses at a multiplicity of infection (MOI) of 20 in the presence of 8 μg/mL polybrene (Genomeditech, China) for 24 h. The cells were then cultured in complete medium for two days and treated with 2.5 μg/mL puromycin (MACKLIN, Shanghai, China) for 7–10 days to establish stable cell lines. Knockdown and overexpression efficiencies of CLDN4 were assessed by Western blotting.

**TABLE 3:** shRNA sequences targeting CLDN4.

shRNA	Target sequences (5′ to 3′)
shCLDN4‐#1	5′‐GCTACCTGCTGGTCATCAAGT‐3′
	5′‐ACTTGATGACCAGCAGGTAGC‐3′
shCLDN4‐#2	5′‐GGTGAAGTACATGGCCTTCGT‐3′
	5′‐ACGAAGGCCATGTACTTCACC‐3′
shCLDN4‐#3	5′‐CCATCACCGTGGTCACTTTGA‐3′
	5′‐TCAAAGTGACCACGGTGATGG‐3′

### 2.6 Transient transfection of miRNA mimics

A total of 5 × 10^5^ B‐CPAP cells were seeded in 60 mm plates 18–24 h prior to transfection. Synthetic miRNA mimics (General Biol) were transfected into cells following the Lipofectamine 3000 transfection protocol. Twenty‐four hours later, cells were split into two 60 mm plates in antibiotics‐containing media and cultured for an additional 48 h.

A synthetic inhibitor of hsa‐miR‐941 obtained from General Biol, we sent it into cells with Lipofectamine 3000 (from Invitrogen, California, USA) according to the instructions. At the same time, we also used the same method and the same concentration, and delivered the same Mission Synthetic Negative Control from General Biol into the cells through the same transfection reagent.

### 2.7 RNA extraction and quantitative real‐time RT‐PCR

We used TRIzol Reagent (Invitrogen, USA) to extract Total RNA from the sample according to the instructions. Then, according to the operating instructions, we synthesized complementary DNA (cDNA) from RNA by using Prime Script RT Reagent Kit (RR 037 Q, Takara, Japan). We did Quantitative PCR on Quantum Studio 6 Flex System (Applied Biosystems, USA). The reagent used was Sybr Green Master Mix (Roche, Switzerland), and each sample was repeated three times. Finally, we calculated the relative expression of the gene by 2^−ΔΔCt^ method.

Total RNA was extracted from the samples and reverse‐transcribed into cDNA using the Mirna First‐Strand cDNA Synthesis Kit (Vazyme, #MR101). Subsequently, the expression levels of miRNAs were quantified by RT‐qPCR with miRNA Universal SYBR QPCR Master Mix (Vazyme, #MR101‐01). The relative expression was calculated using the 2^−ΔΔCt^ method with miRNA U6 as the endogenous control. All primers used in this study were synthesized by General Biol (Chuzhou, China), and their specific sequences are listed in Table 4.

**TABLE 4:** Primers sequence.

Primer	Sequences (5′ to 3′)
Human‐CLDN4‐F	AGTGCAAGGTGTACGACTCGCT
Human‐CLDN4‐R	CGCTTTCATCCTCCAGGCAGTT
Hsa‐miR‐941‐F	TAGGTAGAATGAGGATGCTGT
Hsa‐miR‐941‐R	GTGCAGGGTCCGAGGTATT
Human‐GAPDH‐F	GTCTCCTCTGACTTCAACAGCG
Human‐GAPDH‐R	ACCACCCTGTTGCTGTAGCCAA
Human‐U6‐F	CTCGCTTCGGCAGCACA
Human‐U6‐R	AACGCTTCACGAATTTGCGT

### 2.8 Western blotting

We extracted protein and did western blotting according to the method previously determined.^22^ Cells and thyroid tissue samples of each experimental group were put in RIPA buffer (P0045, Beyotime, Wuhan, China) and mashed at 4 C for 30 min. We used the BCA test kit (P0012, Beyotime) to measure the concentration of protein. Then put the same amount of protein onto a 10% SDS‐PAGE gel to run electrophoresis, and then transferred it to a PVDF membrane (FFP39, Beyotime). We used the following primary antibodies for immunoblotting: anti‐cldn4 (16195‐1‐AP, 1: 500, Proteintech), anti‐P‐PI3K (4292s, 1: 1000, CST), anti‐P‐Akt (66444‐1‐ig, 1:1000, Proteintech). Anti‐PTEN (60300‐3‐IG, 1: 3000, Proteintech) and Anti‐GAPDH (60004‐1‐IG, 1: 50000, Proteintech). The antigen combined with the primary antibody was incubated with the secondary antibody (SA00001‐2, Proteintech) labeled with HRP, and the dilution ratio was 1:10,000. We used enhanced chemiluminescence substrate (P0018HS, Beyotime) to develop the strip. Finally, we used ImageJ software (V1.8.0.112) to measure the gray value of the strip.

### 2.9 Dual‐luciferase reporter assay

To investigate the potential binding sites of miR‐941 on CLDN4, we utilized TargetScan for prediction and amplified the 3′‐untranslated region (3′ UTR) of CLDN4, including both wild‐type and mutant variants, by PCR, followed by cloning into the pmirGLO vector (E1330, Promega, WI, USA). We also changed the predicted binding site of miR‐941 by site‐directed mutation, and then integrated the mutated sequence into the pmirGLO vector by restriction endonuclease and ligase, thus obtaining the mutant dual luciferase reporter gene construct. Twenty‐four hours after transfection, we used the Dual‐Luciferase detection kit (E1910, Promega) to measure luciferase activity.

### 2.10 CCK8 assay

Cell proliferation was measured using a WST‐8 Kit (CCK‐8; Dojindo, Tokyo, Japan). Cells (1 × 10^4^ cells/well) were cultured in a 96‐well plate with 100 μL DMEM containing 10% FBS. CCK‐8 (10 μL/well) was added and incubated at 37°C for 3 h. The absorbance was detected at the 450 nm wavelength with a microplate reader (SpectraMax Paradigm, Molecular Devices, USA). All assays were repeated at least 3 times.

### 2.11 Wound healing assay

The cells were cultured in 6‐well plates until they grew to confluence in 10% FBS medium. Then, the monolayer of cells was scratched with a pipette tip and cultured in serum‐free medium. Migration was photographed under an inverted microscope (MIX60‐FL, Mshot, Guangzhou, China) and quantified by measuring scratch width at 0, 12, and 24 h post‐wounding.

### 2.12 Colony formation assay

We put the cells in a standard laboratory environment, then take them down with trypsin, wash them with PBS, count how many there are, and then plant them in 6‐well plates, with 200 cells in each well. We repeated the experiment three times. All the samples were cultured for 14 days, so that clones consisting of at least 50 cells could grow. After the culture, we stained these clones with crystal violet solution. We counted the clones of qualified size and took some representative photos. Finally, these clones were analyzed by ImageJ software.

### 2.13 Transwell migration assay

Cells were plated in a Transwell chamber (BD Biosciences) with a density of 1 × 10^4^ cells per well.^23^ The lower layer of the 24‐well plate was filled with standard cell culture medium. After 24 h of culture, we used cotton swabs to remove the cells that remained on the upper layer of the membrane and did not pass through. Then, these chambers were fixed with 4% methanol, dyed with 0.1% crystal violet solution at room temperature for 3–5 min, and finally photographed under a microscope to obtain representative images.

### 2.14 Apoptosis assay

After transfection, we cultured the cells in a 6‐well plate for 24 h. When the time was up, we collected the cells and suspended them in a 500 μL binding buffer. In order to see whether the cells have undergone apoptosis, we stained the cells with propidium iodide (PI) and FITC‐Anti‐Annexin V Antibody (HY‐K1073, MCE, Shanghai, China) at room temperature in the dark environment for 15 min. We used a Beckman Coulter Flow Cytometer (USA) to do the flow cytometry analysis, and the data were obtained and analyzed by the software CellQuest Software v3.3.

### 2.15 Transwell co‐culture assay for macrophage polarization

THP‐1 cells were differentiated into macrophages by treatment with 100 ng/mL phorbol 12‐myristate 13‐acetate (PMA, P8139, Sigma) for 48 h, followed by resting in PMA‐free medium for 24 h. B‐CPAP cells (5 × 10^4^ cells/well) stably overexpressing or knocking down CLDN4, along with their negative controls, were seeded into the lower chamber of 24‐well Transwell plates (Corning, 0.4 μm pore size) in complete DMEM with 10% FBS and allowed to adhere overnight. Differentiated macrophages (2 × 10^5^ cells/well) were then added to the upper chamber in serum‐free RPMI‐1640 and co‐cultured for 48 h. After co‐culture, macrophages were collected and stained with APC‐anti‐CD45 (BioLegend), FITC‐anti‐CD86 (BioLegend), and PE‐anti‐CD206 (BioLegend). Cells were gated on the CD45^+^ population, and the proportions of M1 (CD45^+^CD86^+^) and M2 (CD45^+^CD206^+^) macrophages were determined by flow cytometry.

### 2.16 In vivo tumor model

In vivo tumor model: The Institutional Animal Ethics Committee granted approval for the animal research protocol (No. SL202601001). Female BALB/c Nude Mice were housed in a specific pathogen‐free environment. All animals had free access to feed and water, under standardized conditions (12L:12D cycle). Briefly, female BALB/c nude mice were randomly allocated into five groups before inoculation: untransfected Control, OE‐NC, OE‐CLDN4, KD‐NC, and KD‐CLDN4. The stably transfected B‐CPAP cells were subcutaneously injected into the left axilla of each mouse. Tumor length (L) and width (W) were measured with a digital caliper every 2 days, and tumor volume was calculated using the formula V = (L × W^2^)/2. Once tumors reached 100–200 mm^3^, mice bearing OE‐NC or OE‐CLDN4 tumors were each further randomized into two subgroups receiving either LY294002 (50 mg/kg, i.p., every 48 h) or an equivalent volume of vehicle, thereby generating the OE‐NC, OE‐NC + LY294002, OE‐CLDN4, and OE‐CLDN4 + LY294002 groups (*n* = 5 per group). Mice were monitored until a predetermined experimental endpoint, and any mouse whose tumor volume exceeded 1000 mm^3^ was euthanized earlier for humane reasons. At the endpoint, all mice were euthanized, and subcutaneous xenograft tumors were excised for gross imaging and immunohistochemical analysis.

### 2.17 Histopathology

Tumor tissues obtained from clinical and mouse models were preserved in 10% neutral formalin and then embedded in paraffin. We prepared a cross‐section with a thickness of 5 microns and mounted it on a glass slide with an adhesive coating. After that, the tissue sections were dewaxed and stained with H&E (C0105M, Beyotime) according to the manufacturer's instructions.

For IHC staining, paraffin sections were processed; antigen was retrieved by incubating the slides in EDTA buffer at 95°C for 15 min, peroxidase was blocked with 3% H_2_O_2_, and then with 3% bovine serum albumin. Next, the tissue sections were incubated overnight at 4°C with the primary antibody in a humidified chamber. The following primary antibodies and dilutions were used: CLDN4 (16195‐1‐AP, 1:100, Proteintech) and Ki67 (1:2000, 28074‐1‐AP, Proteintech). The samples were then subjected to another incubation using 1:500 diluted horseradish peroxidase‐conjugated goat anti‐rabbit antibodies (1:500, SA00004‐2, Proteintech) as secondary antibodies. Histological images were captured with an OLYMPUS DP 72 camera attached to the microscope.

### 2.18 Statistical analysis

We used GraphPad Prism 9.0 (GraphPad Software, San Diego, CA) for statistical calculations. All the results are expressed as mean ± SEM. For determining statistical significance, we used a *t*‐test when comparing two groups of data and used ANOVA adjusted by Bonferroni when comparing many groups of data. If the *p*‐value of a result is less than 0.05, we consider it statistically significant.

## 3 RESULTS

### 3.1 Single‐cell transcriptomic landscape of PTC

Integrated analysis of scRNA‐seq data from six paired PTC and adjacent normal thyroid samples identified a total of ten major cell populations based on canonical marker gene expression (Figure 1A,B), including B cells, plasma cells, T cells, macrophages, neutrophils, plasmacytoid dendritic cells (pDCs), fibroblasts, endothelial cells, lymphatic endothelial cells, and epithelial cells. Comparative cell proportion analysis between tumor and normal tissues revealed marked alterations in immune and epithelial compartments (Figure 1C). T cells consistently accounted for a higher proportion in PTC tissues than in matched normal samples across all patients, suggesting an immunosuppressive tumor microenvironment. In contrast, macrophages were more abundant in normal tissues compared with tumor tissues in most patients, with the exception of PTC5. Epithelial cells exhibited an increased proportion in tumor tissues relative to normal counterparts in all patients except PTC5, indicating tumor‐associated epithelial expansion.

**FIGURE 1:**
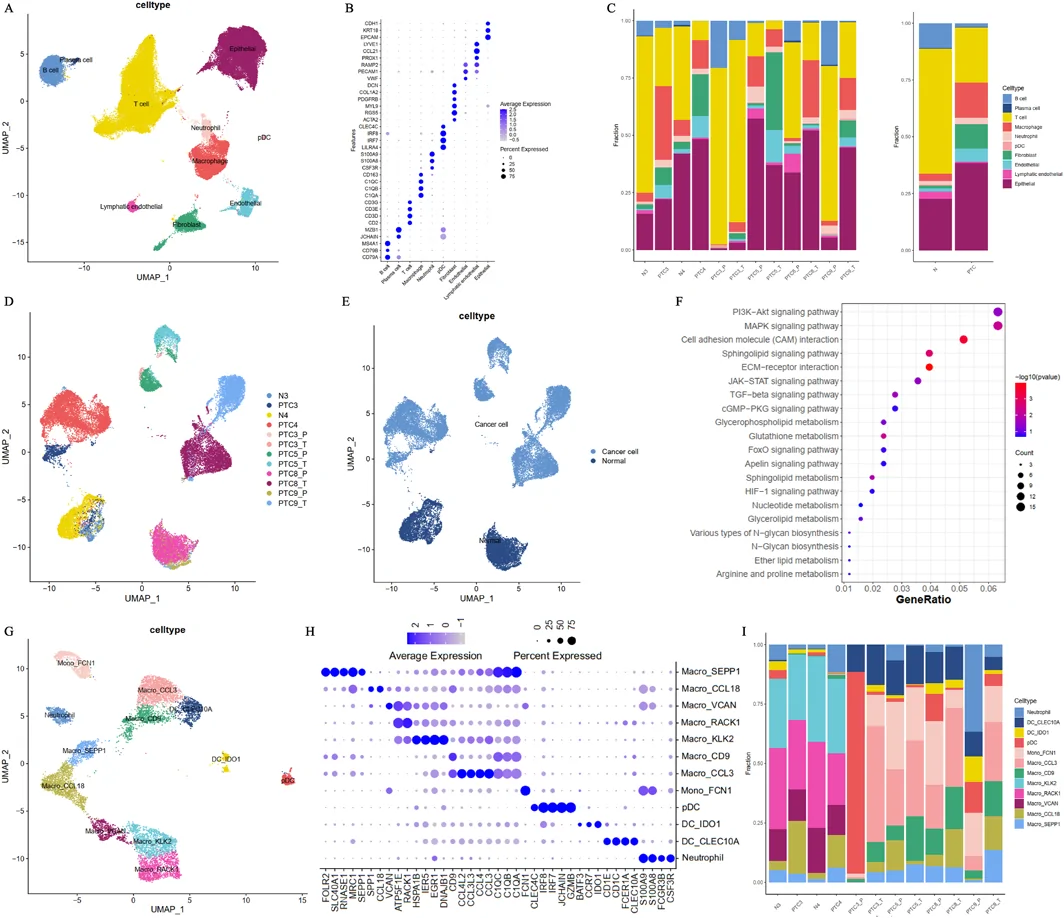
Single‐cell transcriptomic data of papillary thyroid carcinoma (PTC) identified 10 cell types. (A) Integrated UMAP plot of all samples, showing the dimensionality reduction and clustering of cells. (B) Dot plot displaying the expression of canonical cell type markers across the identified cell types. (C) Left panel: Bar plot illustrating the proportion of each cell type within individual samples. Right panel: Bar plot showing the proportion of each cell type across experimental groups. (D) UMAP plot of epithelial cells, with colors representing different samples. (E) UMAP plot of epithelial cells, with colors representing distinct cell subclusters or types. (F) Dot plot of KEGG pathway enrichment analysis comparing tumor cells vs. normal epithelial cells. (G) UMAP plot of the myeloid cell compartment, showing its subclustering. (H) Dot plot exhibiting the expression of signature genes for each myeloid subpopulation. (I) Bar plot quantifying the proportion of each myeloid subpopulation within every sample.

Given the central role of epithelial cells in tumorigenesis, epithelial cells were extracted for further subclustering analysis. UMAP visualization demonstrated a clear separation between normal epithelial cells and tumor epithelial cells, indicating substantial transcriptional heterogeneity between the two populations (Figure 1D,E). Differential gene expression and pathway enrichment analyses revealed that tumor epithelial cells were significantly enriched in pathways related to PI3K–Akt signaling, MAPK signaling, and cell adhesion–associated processes (Figure 1F), implicating these pathways in PTC epithelial malignant transformation. In addition, detailed subclustering analysis of the myeloid compartment identified twelve distinct myeloid cell subpopulations (Figure 1G,H). Notably, a CCL18‐expressing macrophage subpopulation (Macro_CCL18) was detected in all PTC samples and consistently exhibited a higher proportion in tumor tissues compared with matched normal tissues, except in PTC5 (Figure 1I). This finding suggests a potential tumor‐associated macrophage subset involved in PTC progression.

### 3.2 Integrative bulk and single‐cell transcriptomic analysis identify CLDN4 as a progression‐associated epithelial gene in PTC

To identify epithelial‐specific genes associated with PTC, differential expression analysis was first performed using TCGA‐THCA bulk RNA‐seq data by comparing tumor tissues with matched normal thyroid tissues (Figure 2A). To enhance specificity and reduce confounding effects from stromal and immune components, differentially expressed genes were intersected with epithelial cell–specific genes identified from the scRNA‐seq dataset. This integrative analysis yielded four epithelial‐enriched differentially expressed genes: MGST1, NKX2‐1, CLDN4, and CLDN7 (Figure 2A,B). Subsequent comparison of gene expression between tumor epithelial cells and normal epithelial cells revealed that CLDN4 and MGST1 were significantly upregulated in tumor epithelial cells, whereas NKX2‐1 and CLDN7 did not display consistent tumor‐specific elevation (Figure 2C). To evaluate the clinical relevance of these candidate genes, their expression levels were correlated with pathological tumor stage in the TCGA‐THCA cohort. Notably, only CLDN4 expression exhibited a significant positive correlation with advanced tumor stage, whereas the other genes showed no significant stage‐dependent differences (Figure 2D), highlighting CLDN4 as a potential progression‐associated biomarker in PTC.

**FIGURE 2:**
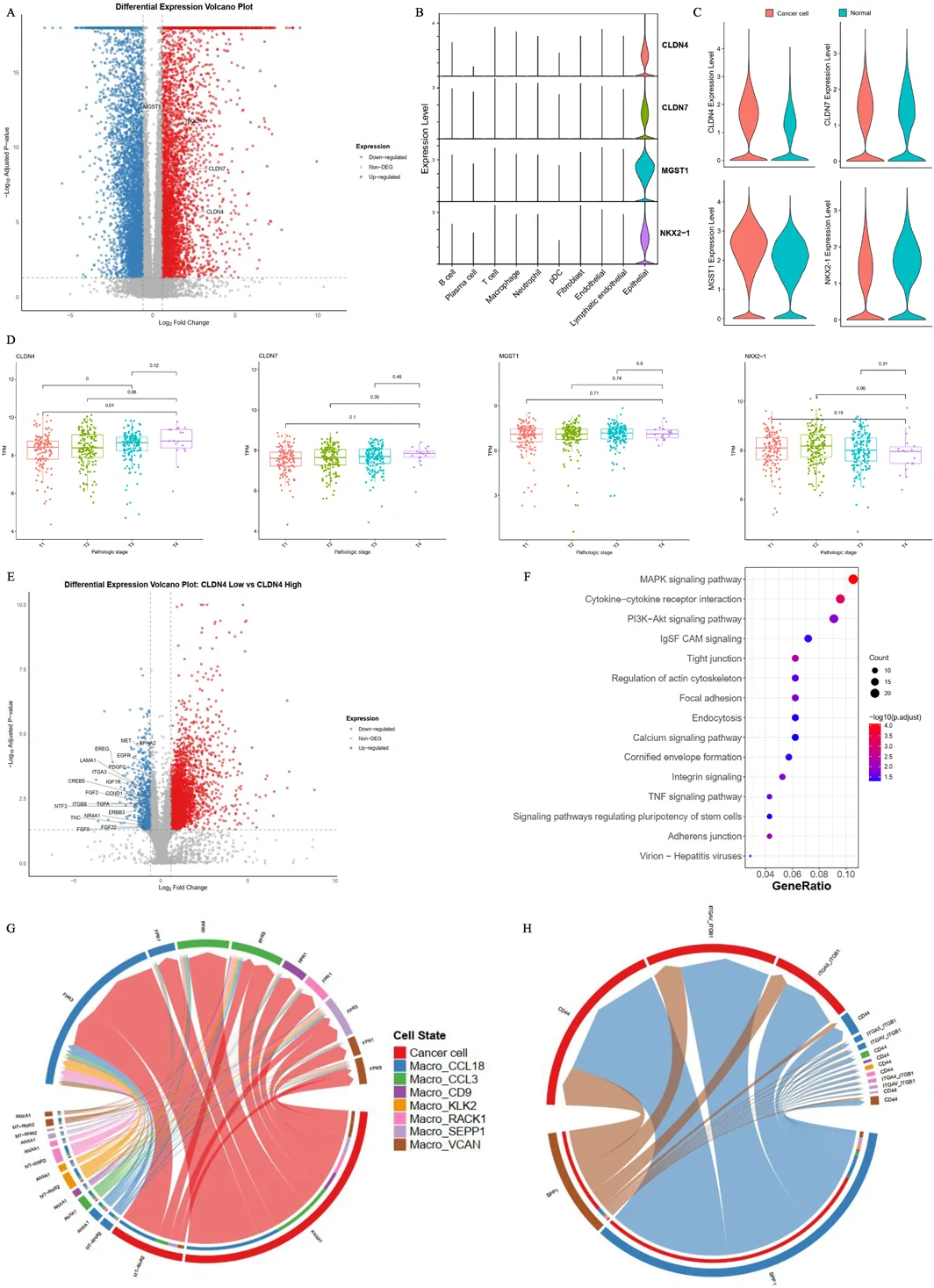
Integrated analysis of bulk and single‐cell RNA‐seq identifies CLDN4 as a biomarker significantly associated with PTC progression and stage. (A) Volcano plot of DEGs from TCGA‐THCA bulk RNA‐seq data, comparing tumor vs. normal tissues. Four genes specifically expressed in epithelial cells within the single‐cell dataset are highlighted. (B) Violin plots showing the expression levels of the four highlighted genes across various cell types identified in the single‐cell dataset. (C) Violin plots comparing the expression of the four genes between tumor cells and normal epithelial cells, revealing significantly higher expression of CLDN4 and MGST1 in tumor cells. (D) Box plots illustrating the association between the expression of these genes and pathological tumor stage. Only CLDN4 expression shows a significant correlation with advanced stage. (E) Volcano plot of DEGs from TCGA THCA bulk RNA‐seq data, comparing samples with low vs. high CLDN4 expression. Genes belonging to the PI3K‐AKT signaling pathway are annotated. (F) KEGG pathway enrichment analysis of upregulated genes in CLDN4‐high vs. CLDN4‐low samples, highlighting significant enrichment of the PI3K‐AKT signaling pathway. (G) Interaction plot depicting ligand‐receptor pairs within the SPP1 signaling pathway between tumor cells and macrophage subsets. (H) Interaction plot depicting ligand‐receptor pairs within the ANNEXIN signaling pathway between tumor cells and macrophage subsets.

Based on these findings, TCGA‐THCA samples were stratified into CLDN4‐high and CLDN4‐low groups according to median CLDN4 expression. Differential gene expression analysis between the two groups identified a distinct transcriptional signature associated with high CLDN4 expression (Figure 2E). KEGG pathway enrichment analysis of upregulated genes in the CLDN4‐high group revealed significant enrichment of the PI3K–AKT signaling pathway (Figure 2F), suggesting that CLDN4 may be functionally linked to PI3K–AKT pathway activation in PTC. To further explore the interactions between CLDN4‐high tumor epithelial cells and the tumor immune microenvironment, cell–cell communication analysis was performed focusing on epithelial and myeloid cell compartments. CellChat analysis revealed prominent ligand–receptor interactions between tumor epithelial cells and macrophage subsets. Specifically, SPP1 expressed by tumor cells interacted with CD44 on the Macro_CCL18 macrophage subpopulation, potentially promoting tumor cell proliferation (Figure 2G). Conversely, Macro_CCL18‐regulated tumor cells exhibited enhanced ANXA1–FPR3 signaling, which may contribute to immune evasion within the tumor microenvironment (Figure 2H). Collectively, these findings indicate that CLDN4‐high tumor epithelial cells are closely associated with PI3K–AKT pathway activation and engage in reciprocal pro‐tumorigenic communication with CCL18^+^ macrophages.

### 3.3 CLDN4 is highly expressed in thyroid cancer tissues

To validate the clinical relevance of our transcriptomic findings, we first examined *CLDN4* expression in a cohort of 25 paired thyroid cancer and adjacent non‐tumor tissues. qRT‐PCR analysis revealed a significant upregulation of *CLDN4* mRNA in tumor tissues compared with matched normal counterparts (Figure 3A). Conversely, the expression of miR‐941, identified as an upstream regulator of CLDN4, was markedly downregulated in the same set of tumor samples (Figure 3B), suggesting an inverse correlation between miR‐941 and CLDN4 in thyroid cancer. At the protein level, Western blot analysis of five representative paired samples confirmed a consistent increase in CLDN4 protein expression in tumor tissues relative to adjacent normal tissues (Figure 3C). Immunohistochemical staining further substantiated this finding, showing stronger CLDN4 immunoreactivity in thyroid carcinoma tissues compared with their paracancerous counterparts (Figure 3D). To extend these observations beyond our institutional cohort, we analyzed CLDN4 expression using the GEPIA database, which includes 512 thyroid tumor and 337 normal thyroid tissues. This external validation demonstrated a statistically significant elevation of CLDN4 in tumor samples (Figure 3E). We next evaluated the clinical significance of CLDN4 expression by stratifying 82 thyroid cancer patients into low CLDN4 (*n* = 19) and high CLDN4 (*n* = 63) groups based on the average CLDN4 expression level in tumor tissues. Clinicopathological analysis revealed that high CLDN4 expression was significantly associated with larger tumor size (≥3.2 cm, *p* = 0.0003), lymphatic metastasis (*p* = 0.0064), distant metastasis (*p* = 0.0405), and vascular invasion (*p* = 0.0093). However, no significant correlations were observed between CLDN4 expression and gender, age, TNM staging, distant metastasis, recurrence, or death (Table 5). These findings indicate that elevated CLDN4 expression is closely associated with aggressive clinicopathological features in thyroid cancer, further supporting its role as a progression‐related biomarker.

**FIGURE 3:**
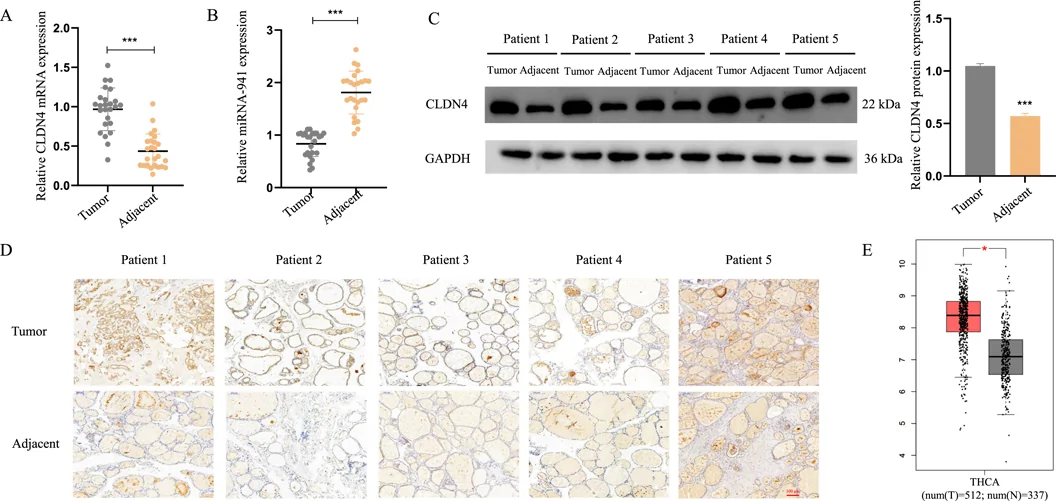
CLDN4 expression is significantly upregulated in clinical thyroid cancer tissue samples. (A) qRT‐PCR analysis of *CLDN4* mRNA expression in 25 paired thyroid cancer tissues (Tumor) and adjacent non‐tumor tissues (Adjacent). (B) qRT‐PCR analysis of miR‐941 expression in the same set of 25 paired clinical samples. (C) Western blot analysis of CLDN4 protein expression in five representative paired thyroid cancer (T) and adjacent normal (N) tissue samples. GAPDH served as a loading control. (D) Representative IHC staining of CLDN4 in paired thyroid cancer and adjacent normal tissues from five patients (scale bar = 100 μm). (E) Box plot showing CLDN4 expression levels in thyroid cancer from the GEPIA database, comparing 512 tumor tissues (red) and 337 normal thyroid tissues (black). Data are presented as mean ± SEM. ***p < 0.001.

**TABLE 5:** Correlation between CLDN4 expression and clinicopathological characteristics in thyroid cancer patients.

Variable	Total (*n* = 82)	Low CLDN4 (*n* = 19)	High CLDN4 (*n* = 63)	*p*‐value
Gender				
Male	8	3	5	0.3119
Female	74	16	58	
Age (years)				
<62	23	5	18	0.8479
≥62	59	14	45	
Size (cm)				
<3.2	28	13	15	0.0003*
≥3.2	54	6	48	
Lymphatic metastasis				
Yes	44	5	39	0.0064**
No	38	14	24	
Distant metastasis				
Yes	24	2	22	0.0405*
No	58	17	41	
Vascular invasion				
Yes	51	7	44	0.0093**
No	31	12	19	
Recurrence				
Yes	41	11	30	0.4323
No	41	8	33	
Death				
Yes	11	2	9	0.6734
No	71	17	54	

*p < 0.05; **p < 0.01.

### 3.4 CLDN4 promotes proliferation, migration, and suppresses apoptosis in B‐CPAP cells

Building on findings of aberrant CLDN4 expression, the baseline CLDN4 level was evaluated across a panel of thyroid cancer cell lines. Compared with the normal thyroid follicular epithelial cell line Nthy‐ori 3‐1, all four PTC cell lines (TPC‐1, B‐CPAP, MDA‐T85, and K1) exhibited significantly elevated CLDN4 mRNA expression (Figure 4A, all *p* < 0.001). Among them, B‐CPAP cells displayed the most pronounced upregulation, with CLDN4 transcription reaching approximately 65‐ to 70‐fold higher than that of Nthy‐ori 3‐1, followed by TPC‐1 cells. Therefore, B‐CPAP cells, which showed the highest CLDN4 expression, were selected as the primary model for the subsequent functional and mechanistic experiments, whereas TPC‐1 cells were reserved for later validation of the key findings in an independent PTC genetic background.

**FIGURE 4:**
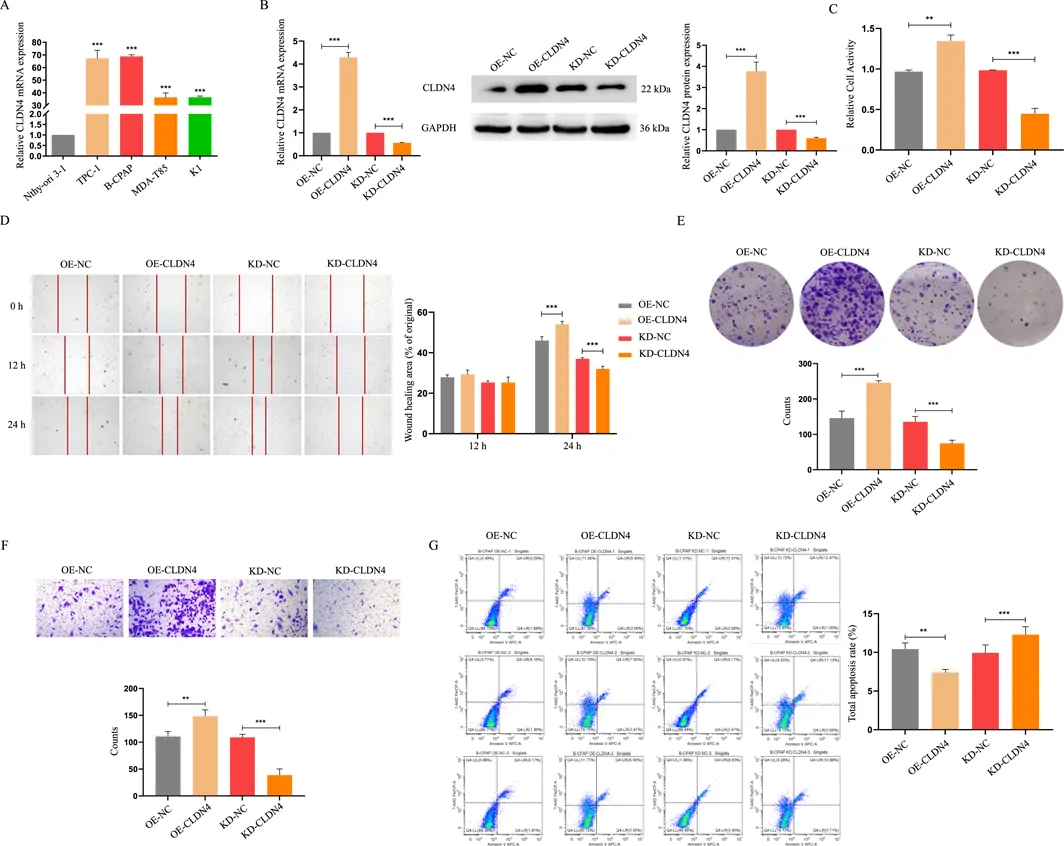
CLDN4 promotes proliferation, migration, and suppresses apoptosis in B‐CPAP cells. (A) The mRNA levels of CLDN4 in the Nthy‐ori 3‐1, TPC‐1, B‐CPAP, MDA‐T85, and K1 cells were measured by qRT‐PCR. (B) The mRNA and protein expression of CLDN4 in OE‐CLDN4, OE‐NC, KD‐NC, and KD‐CLDN4 were measured by qRT‐PCR and western blot to evaluate the transfection efficiency of CLDN4. (C) CCK‐8 assay assessing proliferation of B‐CPAP cells following CLDN4 transfection. (D) Wound healing assay assessing CLDN4‐mediated migration in B‐CPAP cells (magnification, ×10). (E) Assessment of proliferative capacity in CLDN4‐ transfected B‐CPAP cells by clonogenic assay (magnification, ×20). (F) Evaluation of CLDN4‐mediated cell migration through Transwell chamber system (magnification, ×20). (G) Flow cytometric evaluation of apoptosis in CLDN4‐modified B‐CPAP cells. Data are presented as mean ± SEM from three independent experiments. *p < 0.05, **p < 0.01, ***p < 0.001.

To investigate the functional role of CLDN4, we generated stable CLDN4‐overexpressing (CLDN4‐OE) and CLDN4‐knockdown (CLDN4‐KD) B‐CPAP cells via lentiviral transduction. For knockdown, we used three independent shRNA sequences targeting CLDN4 (shCLDN4‐#1, #2, #3). Transfection efficiency was validated by western blotting and qPCR, respectively (Figure 4B), and the knockdown efficacy of each shRNA is presented in Supporting Information S1: Figure S1.

Assessed by CCK‐8 assay, CLDN4‐OE significantly promoted B‐CPAP cell proliferation, while its knockdown markedly suppressed cell viability (Figure 4C). Both wound healing and transwell assays demonstrated that the overexpression of CLDN4 significantly enhanced the migratory capacity of B‐CPAP cells, whereas CLDN4 knockdown had an inhibitory effect on cell migration (Figure 4D,F). In addition, the plate colony formation assay, another assay to examine cell proliferation, further confirmed the enhanced effect of increased CLDN4 expression on B‐CPAP cell proliferation, while CLDN4 knockdown suppressed the colony formation ability in cells (Figure 4E). Finally, flow cytometry revealed that CLDN4‐OE diminished B‐CPAP cell apoptosis, whereas CLDN4‐KD accelerated apoptotic cell death (Figure 4G). Together, these data thus confirmed that CLDN4 overexpression drives malignant progression in PTC by enhancing oncogenic phenotypes and conferring apoptosis resistance.

### 3.5 miR‐941 directly targets CLDN4 and suppresses CLDN4‐driven malignant phenotypes

To validate whether miR‐941 acts as a direct upstream regulator of CLDN4, we synthesized miR‐941 mimic and inhibitor and transfected them into B‐CPAP cells. Transfection efficiency was confirmed by qRT‐PCR. Compared with the mimic negative control (NC) group, miR‐941 levels were markedly increased in the miR‐941 mimic group. Conversely, compared with the inhibitor NC group, miR‐941 levels were significantly reduced in the miR‐941 inhibitor group (Figure 5A). Western blot analysis showed that CLDN4 protein expression was greatly suppressed in the miR‐941 mimic group, whereas it was significantly enhanced in the miR‐941 inhibitor group (Figure 5B), indicating an inverse regulatory relationship between miR‐941 and CLDN4. To determine whether miR‐941 directly binds to the CLDN4 transcript, we constructed a dual‐luciferase reporter containing the wild‐type (WT) or mutant (MUT) 3‘‐UTR of CLDN4, with six contiguous base pairs within the putative miR‐941 binding site mutated (Figure 5C). Co‐transfection of miR‐941 mimics significantly suppressed the luciferase activity of the CLDN4‐3’UTR‐WT reporter but had no effect on the CLDN4‐3‘UTR‐MUT reporter (Figure 5C), confirming that miR‐941 directly targets the 3’UTR of CLDN4. We next investigated whether miR‐941 exerts its biological functions by regulating CLDN4. To this end, B‐CPAP cells were transfected with CLDN4 overexpression vector alone or in combination with miR‐941 mimics. Functional assays demonstrated that CLDN4 overexpression significantly enhanced cell viability, whereas treatment with miR‐941 mimics markedly reduced cell viability; this inhibitory effect was partially rescued by concurrent CLDN4 overexpression (Figure 5D). Similarly, wound healing assays showed that CLDN4 overexpression promoted cell migration, while miR‐941 mimics suppressed migration, and this effect was reversed by CLDN4 co‐overexpression (Figure 5E). Colony formation assays revealed that CLDN4 overexpression increased proliferative capacity, which was attenuated by miR‐941 mimics and subsequently restored by CLDN4 (Figure 5F). Transwell assays further confirmed that CLDN4‐driven migratory ability was counteracted by miR‐941 mimics and rescued upon CLDN4 re‐expression (Figure 5G). Finally, flow cytometry analysis of apoptosis demonstrated that CLDN4 overexpression reduced apoptotic cell death, whereas miR‐941 mimics increased apoptosis; this pro‐apoptotic effect was reversed by CLDN4 co‐overexpression (Figure 5H). Collectively, these results demonstrate that miR‐941 directly targets CLDN4 and suppresses CLDN4‐driven malignant phenotypes, including proliferation, migration, and apoptosis resistance in thyroid cancer cells.

**FIGURE 5:**
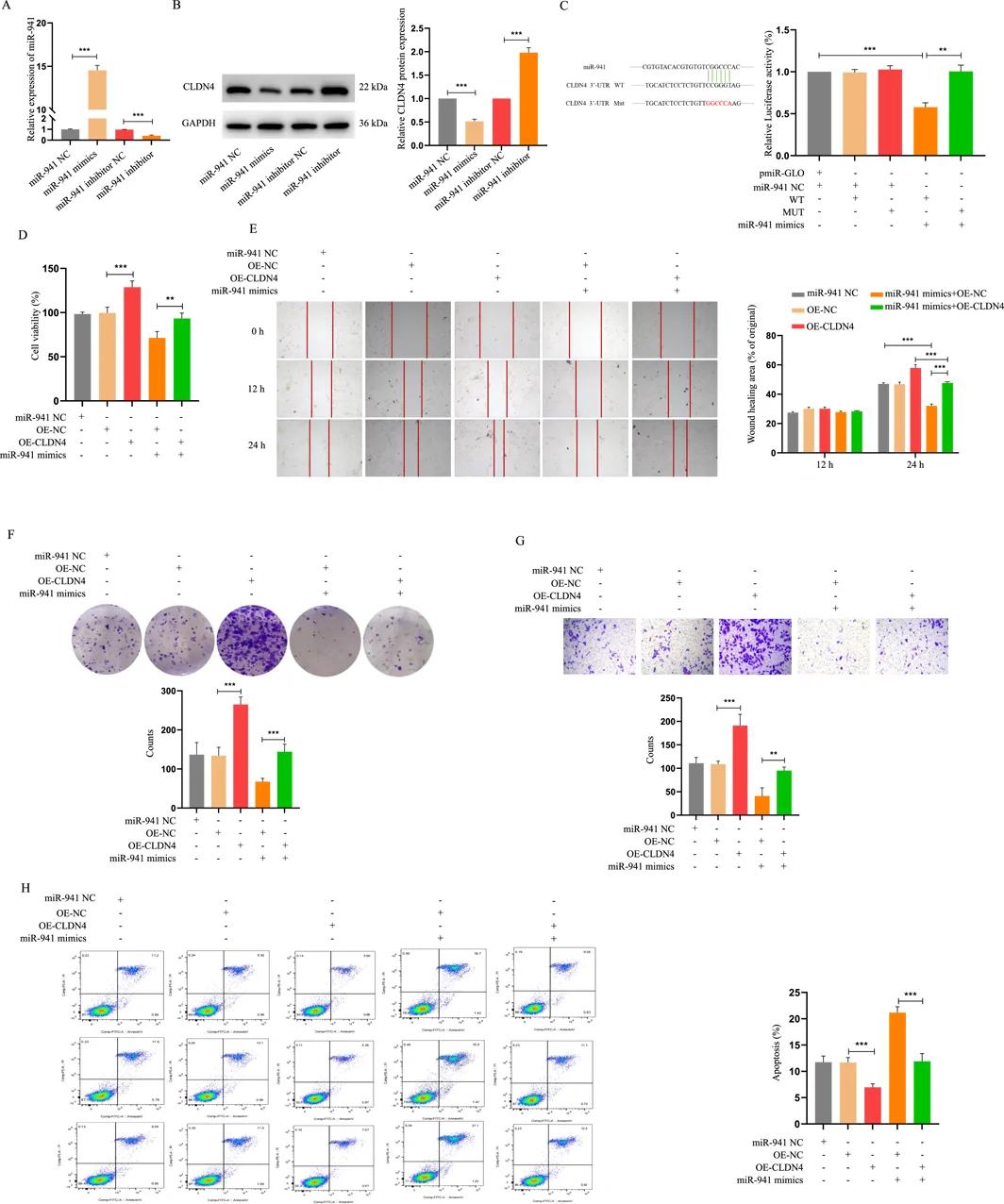
miR‐941 directly targets CLDN4 expression. (A) qRT‐PCR validation of miR‐941 overexpression efficiency following transfection with miR‐941 mimic and knockdown efficiency with miR‐941 inhibitor. (B) Western blot analysis showing CLDN4 protein expression in B‐CPAP cells transfected with miR‐941 mimic or inhibitor. (C) Schematic diagram of the predicted miR‐941 binding site within the CLDN4 3‘‐UTR and the generated mutation (MUT) with six contiguous base pair substitutions. Dual‐luciferase reporter assay showing that miR‐941 mimics significantly suppress the luciferase activity of the CLDN4‐3’UTR‐WT reporter but not the CLDN4‐3‘UTR‐MUT reporter. (D) CCK‐8 assay assessing cell viability in B‐CPAP cells transfected with CLDN4‐OE, miR‐941 mimic, or their combination. (E) Wound healing assay evaluating cell migration under the same conditions (magnification, ×10). (F) Colony formation assay assessing proliferative capacity (magnification, ×20). (G) Transwell assay evaluating migratory ability (magnification, ×20). (H) Flow cytometry analysis of apoptosis. Data are presented as mean ± SEM from three independent experiments. *p < 0.05, **p < 0.01, ***p < 0.001; ns, not significant.

### 3.6 CLDN4 activates the PI3K/AKT pathway, which is reversed by miR‐941

To determine whether CLDN4 exerts its oncogenic effects through the PI3K/AKT pathway, we examined the expression of key pathway components in CLDN4‐modulated B‐CPAP cells. Western blot analysis showed that CLDN4 overexpression dramatically upregulated phospho‐PI3K (p‐PI3K) and phospho‐AKT (p‐AKT) levels without significantly altering total PI3K or total AKT expression, while PTEN expression was downregulated, indicating specific pathway activation (Figure 6A). Conversely, in CLDN4‐knockdown cells, p‐PI3K and p‐AKT levels were markedly reduced with a concomitant rise in PTEN, whereas total PI3K and total AKT remained unchanged, demonstrating specific inhibition of the PI3K/AKT pathway (Figure 6A). We next investigated whether miR‐941 modulates PI3K/AKT signaling through CLDN4. Transfection of miR‐941 mimics into B‐CPAP cells resulted in significant suppression of the PI3K/AKT pathway, as evidenced by reduced p‐PI3K and p‐AKT levels and increased PTEN expression, without affecting total PI3K or total AKT (Figure 6B). Conversely, the miR‐941 inhibitor led to a rebound in p‐PI3K and p‐AKT levels and a decrease in PTEN expression (Figure 6B). Importantly, the CLDN4 overexpression‐induced activation of PI3K/AKT signaling was effectively reversed upon co‐transfection with miR‐941 mimics, which reduced p‐PI3K and p‐AKT levels and restored PTEN expression (Figure 6B). Collectively, these results indicate that miR‐941 suppresses CLDN4‐driven oncogenicity by attenuating PI3K/AKT pathway activation, and that the observed changes in pathway activity are specific to phosphorylation events rather than alterations in total protein levels.

**FIGURE 6:**
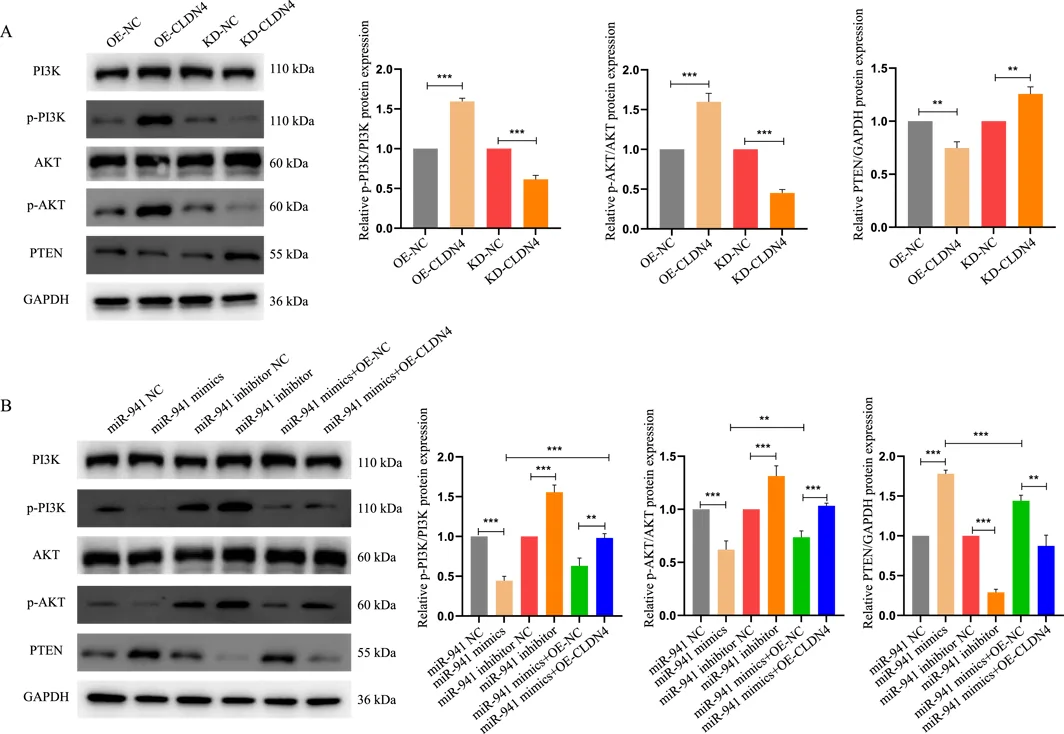
miR‐941 attenuates CLDN4‐induced activation of the PI3K/AKT pathway. (A) Western blot analysis of p‐PI3K, total PI3K, p‐AKT, total AKT, and PTEN protein expression in B‐CPAP stable cell lines with CLDN4 overexpression (OE‐CLDN4) or knockdown (KD‐CLDN4). (B) Western blot analysis of the same panel of proteins in B‐CPAP cells transfected with miR‐941 mimic, miR‐941 inhibitor, or CLDN4‐OE alone or in combination with miR‐941 mimic. GAPDH served as a loading control. Data are presented as mean ± SEM from three independent experiments. *p < 0.05, **p < 0.01, ***p < 0.001; ns, not significant.

### 3.7 CLDN4 promotes malignant phenotypes through PI3K/AKT signaling

To further determine whether CLDN4 exerts its oncogenic effects specifically through the PI3K/AKT pathway, we treated CLDN4‐overexpressing B‐CPAP cells with the PI3K‐specific inhibitor LY294002. Functional assays revealed that LY294002 treatment significantly suppressed cell viability, whereas this inhibitory effect was partially rescued by CLDN4 overexpression (Figure 7A). Wound healing assays demonstrated that LY294002 markedly reduced cell migration, an effect that was reversed upon CLDN4 overexpression (Figure 7B). Colony formation assays showed that LY294002 treatment attenuated proliferative capacity, which was restored by concurrent CLDN4 overexpression (Figure 7C). Transwell assays further confirmed that LY294002 inhibited migratory ability, and this inhibition was counteracted by CLDN4 overexpression (Figure 7D). Collectively, these results demonstrate that CLDN4 promotes malignant phenotypes, including proliferation, migration, and colony formation, through activation of the PI3K/AKT signaling pathway.

**FIGURE 7:**
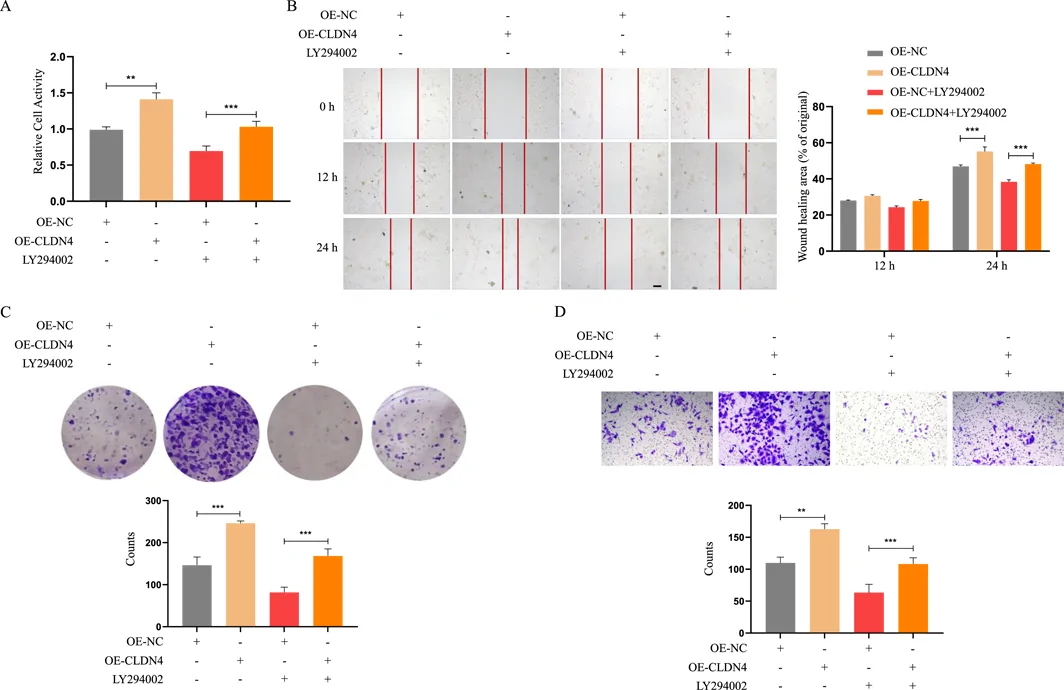
Inhibition of PI3K/AKT signaling by LY294002 suppresses CLDN4‐driven malignant phenotypes in B‐CPAP cells. (A) CCK‐8 assay assessing cell viability in OE‐NC, OE‐CLDN4, OE‐NC + LY294002, and OE‐CLDN4 + LY294002 groups. (B) Wound healing assay evaluating cell migration under the same conditions (magnification, ×10). (C) Colony formation assay assessing proliferative capacity (magnification, ×20). (D) Transwell assay evaluating migratory ability (magnification, ×20). Data are presented as mean ± SEM from three independent experiments. *p < 0.05, **p < 0.01, ***p < 0.001.

### 3.8 miR‐941 suppresses CLDN4‐driven malignant phenotypes in TPC‐1 cells

To validate the generality of the miR‐941/CLDN4 regulatory axis beyond the B‐CPAP cell line, we performed rescue experiments in another PTC cell line, TPC‐1. Cells were transfected with the CLDN4 overexpression vector alone or in combination with miR‐941 mimics, and functional assays were conducted. CCK‐8 assay demonstrated that CLDN4 overexpression significantly enhanced cell viability in TPC‐1 cells, whereas treatment with miR‐941 mimics markedly reduced cell viability. Notably, this inhibitory effect was partially rescued by concurrent CLDN4 overexpression (Figure 8A). Wound healing assays showed that CLDN4 overexpression promoted cell migration, while miR‐941 mimics suppressed migration, and this effect was reversed by CLDN4 co‐overexpression (Figure 8B). Colony formation assays revealed that CLDN4 overexpression increased proliferative capacity, which was attenuated by miR‐941 mimics and subsequently restored upon CLDN4 re‐expression (Figure 8C). Transwell assays further confirmed that CLDN4‐driven migratory ability was counteracted by miR‐941 mimics and rescued by CLDN4 overexpression (Figure 8D). Collectively, these results confirm that the miR‐941/CLDN4 regulatory axis is not limited to B‐CPAP cells but is operative in another PTC model, consistently demonstrating that miR‐941 suppresses CLDN4‐driven malignant phenotypes including proliferation, migration, and colony formation.

**FIGURE 8:**
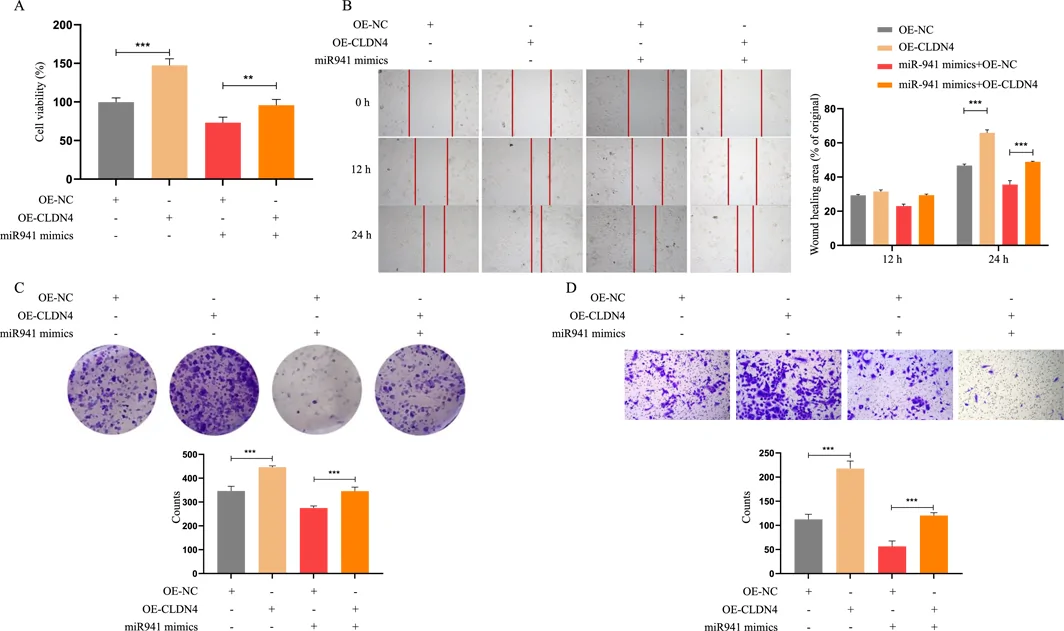
miR‐941 suppresses CLDN4‐driven malignant phenotypes in TPC‐1 cells. (A) CCK‐8 assay assessing cell viability in TPC‐1 cells transfected with OE‐NC, miR‐941 mimics + OE‐NC, or miR‐941 mimics + OE‐CLDN4. (B) Wound healing assay evaluating cell migration at 0, 12, and 24 h under the same conditions (magnification, ×10). (C) Colony formation assay assessing proliferative capacity (magnification, ×20). (D) Transwell assay evaluating migratory ability (magnification, ×20). Data are presented as mean ± SEM from three independent experiments. *p < 0.05, **p < 0.01, ***p < 0.001.

### 3.9 CLDN4 modulates tumor‐associated macrophage polarization

Given our single‐cell transcriptomic analysis revealing potential crosstalk between CLDN4‐high tumor epithelial cells and CCL18^+^ macrophages (Figure 2G,H), together with the established role of CLDN4‐activated PI3K/AKT signaling in regulating macrophage polarization, we further investigated whether CLDN4 expression in thyroid cancer cells influences macrophage polarization. To this end, we co‐cultured THP‐1‐derived macrophages with B‐CPAP cells stably overexpressing or knocking down CLDN4, and assessed M1 and M2 polarization markers by flow cytometry. As shown in Figure 9A, co‐culture with CLDN4‐overexpressing (OE‐CLDN4) tumor cells significantly reduced the proportion of M1‐polarized macrophages, as evidenced by a decreased CD45^+^CD86^+^ cell population compared with the OE‐NC co‐culture group. Conversely, co‐culture with CLDN4‐knockdown (KD‐CLDN4) tumor cells markedly increased the M1 macrophage population, indicating that CLDN4 suppression promotes the anti‐tumor M1 phenotype. In contrast, analysis of M2 polarization revealed the opposite trend (Figure 9B). Co‐culture with OE‐CLDN4 tumor cells significantly enhanced the proportion of M2‐polarized macrophages (CD45^+^CD206^+^), whereas KD‐CLDN4 tumor cells substantially reduced M2 macrophage polarization compared with their respective control groups. These results suggest that CLDN4 expression in thyroid cancer cells promotes M2 polarization while suppressing M1 polarization of tumor‐associated macrophages, suggesting that CLDN4 contributes to an immunosuppressive tumor microenvironment by skewing macrophage polarization toward the pro‐tumor M2 phenotype.

**FIGURE 9:**
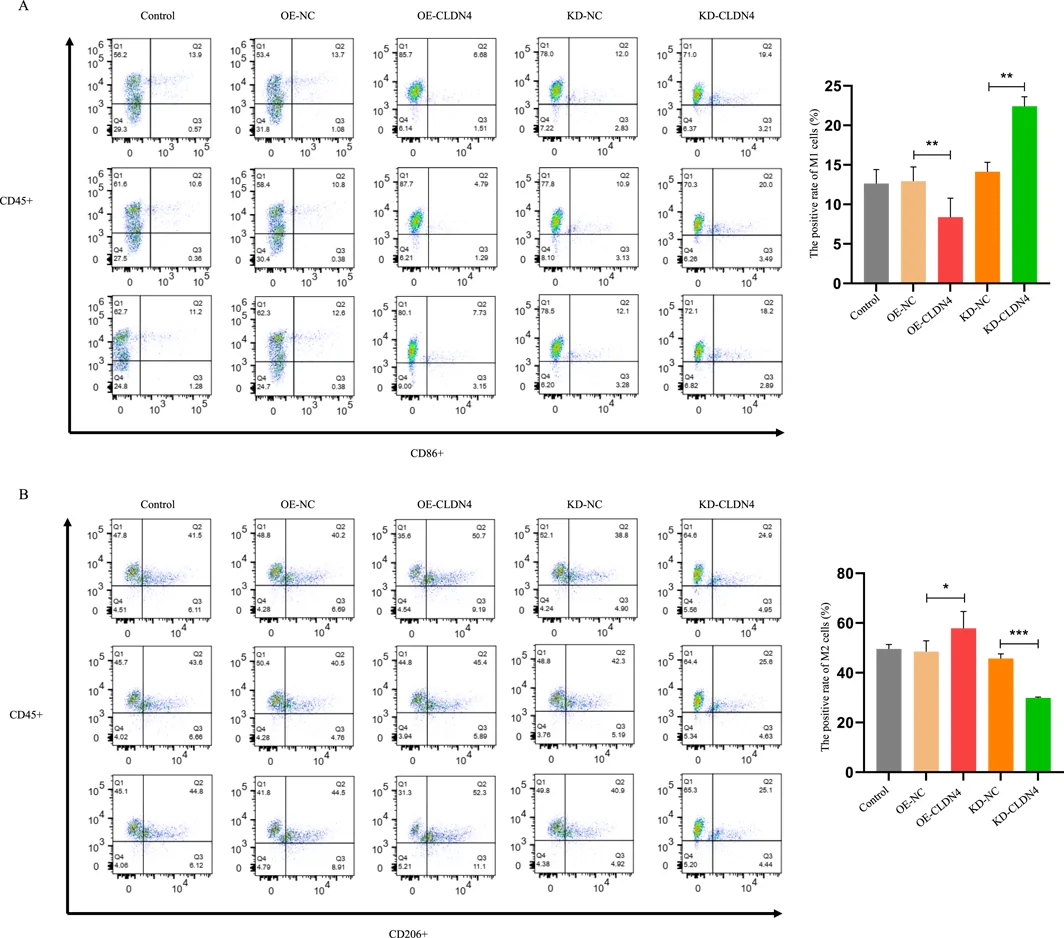
CLDN4 modulates macrophage polarization in co‐culture systems. (A) Flow cytometric analysis of M1 macrophage polarization (CD45^+^CD86^+^) following co‐culture with B‐CPAP cells stably overexpressing (OE‐CLDN4) or knocking down (KD‐CLDN4) CLDN4, compared with respective negative controls (OE‐NC and KD‐NC). (B) Flow cytometric analysis of M2 macrophage polarization (CD45^+^CD206^+^) under the same co‐culture conditions. Data are presented as mean ± SEM from three independent experiments. *p < 0.05, **p < 0.01, ***p < 0.001.

### 3.10 CLDN4 promotes tumor growth via the PI3K signaling in vivo

We extended our investigation to an in vivo setting by employing a xenograft tumor model in immunodeficient mice to assess the physiological function of CLDN4 in tumor proliferation. The PTC tumor models were successfully established by subcutaneous injection of CLDN4‐OE or CLDN4‐KD B‐CPAP cells into the left armpit of BALB/c nude mice. The CLDN4‐OE group exhibited significantly accelerated tumor growth compared to the control group, while the CLDN4‐KD group showed markedly slower tumor progression with observable growth stagnation. Final tumor volumes were approximately twofold larger in the OE‐CLDN4 group than in controls, but were reduced to approximately one‐sixth in the KD‐CLDN4 group. To investigate whether CLDN4 promotes thyroid cancer progression via the PI3K/AKT pathway, we used the PI3K‐specific inhibitor LY294002 in both CLDN4‐OE and control (NC‐OE) groups. The results demonstrated that LY294002 significantly suppressed the promotive effect of CLDN4 on tumor proliferation (Figure 10A,B). Further immunohistochemical analysis of tumor tissues revealed a positive correlation between CLDN4 expression levels and Ki67‐positive rates (Figure 10C,D). The H‐score results demonstrated that the expression of CLDN4 in the OE‐CLDN4 group was significantly higher than that in the control group, while the LY294002‐treated group exhibited significantly lower CLDN4 expression compared to the control (Figure 10C). The trend in Ki67 H‐score across all groups was consistent with that of CLDN4, indicating that CLDN4 acts as an oncogene in thyroid cancer by promoting cell proliferation (Figure 10D). These results collectively demonstrate that CLDN4 significantly promotes tumor progression in vivo, which may be related to the PI3K signaling pathway.

**FIGURE 10:**
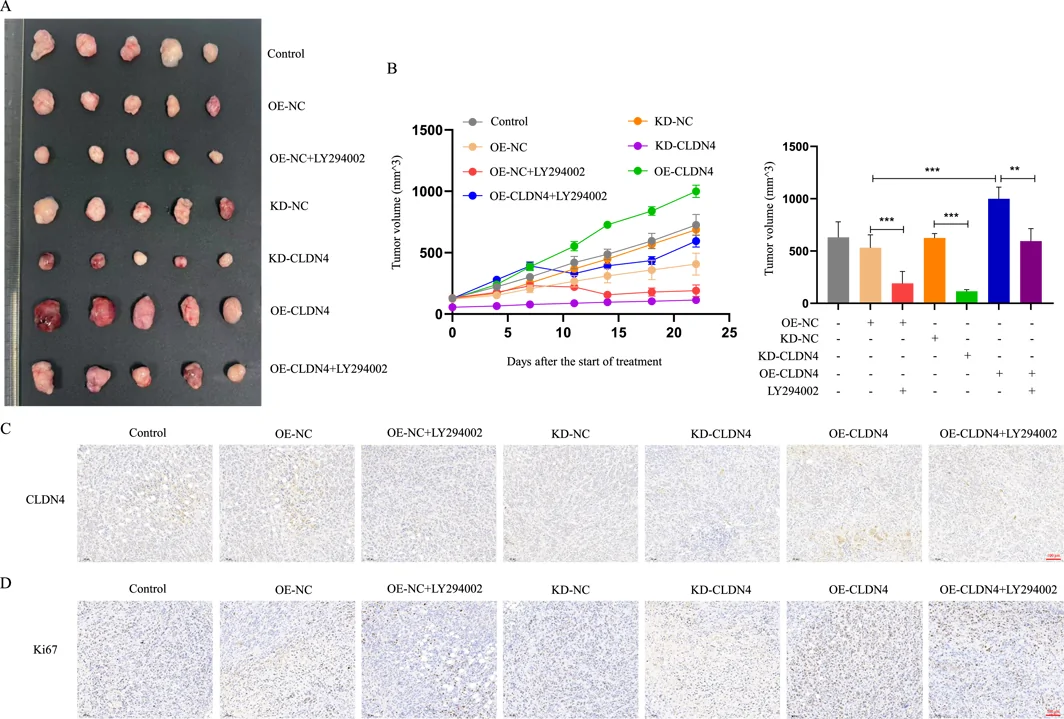
CLDN4 promotes tumor growth in mouse models. (A) Photos of representative excised tumor tissues from different groups, (B) Tumor volume growth curve (left panel) and final tumor volumes at harvest (right panel), (C) Representative images of immunohistochemistry for CLDN4 in mouse tumor tissues from different groups and the H‐score of CLDN4 in each group (10×, bar = 100 μm). (D) Representative images of immunohistochemistry for Ki67 in mouse tumor tissues from different groups and the H‐score of Ki67 in each group (10×, bar = 100 μm).

## 4 DISCUSSION

Globally, thyroid cancer represents the most rapidly increasing malignancy among endocrine system cancers, with PTC being the most common type.^2^ Although the majority of PTC patients exhibit favorable prognoses, a subset develops aggressive features such as local invasion, distant metastasis, or resistance to conventional therapies, leading to disease progression and poor clinical outcomes.^24^ Therefore, elucidating the molecular mechanisms underlying thyroid carcinogenesis and identifying novel diagnostic markers and therapeutic targets remain critically important. A comprehensive analysis of clinical samples and database resources indicates that CLDN4 may be a potential driver of thyroid cancer. Based on this finding, the tumor‐promoting effect of CLDN4 was investigated using both in vitro and in vivo thyroid cancer models, providing new theoretical insights into the progression mechanism of this malignancy.

This study elucidates the biological function and underlying regulatory mechanism of CLDN4 in thyroid cancer. Based on comprehensive analyses of clinical specimens, cellular models, and in vivo experiments, we have validated its significant oncogenic role in thyroid carcinogenesis. As a key tight junction protein, CLDN4 dysfunction has been closely related to tumor development.^25^, ^26^ This study shows that CLDN4 protein expression is significantly upregulated in thyroid cancer, consistent with its expression patterns in gastric cancer, high‐grade serous ovarian cancer, cervical cancer, glioma, and other malignancies.^27^, ^28^, ^29^, ^30^, ^31^ In addition, the consistent elevation of CLDN4 expression and its positive correlation with the Ki67 proliferation index support its potential as both a diagnostic and prognostic biomarker for thyroid cancer. Furthermore, our results provide compelling theoretical rationale and preclinical evidence for therapeutic targeting of CLDN4. Notably, ASP1002, a dual‐targeting bispecific antibody against CLDN4 and 4‐1BB, has advanced to Phase I clinical trials for CLDN4‐positive solid tumors (NCT05719558).^14^ CLDN4 is a key tight junction protein that primarily maintains the barrier function of epithelial and endothelial cells under physiological conditions. In tumor tissues, however, CLDN4 can promote EMT, maintain the characteristics of tumor stem cells, and greatly enhance the cell proliferation and migration capacity through activation of critical signaling pathways such as integrin β1/YAP, Src/AKT, and PI3K/AKT.^27^, ^32^, ^33^

CLDN4 acts as a pivotal regulator of EMT; for instance, it facilitates cervical cancer metastasis via Twist1‐mediated transcriptional activation.^28^ It also plays a key role in maintaining cancer stemness. The functional importance of CLDN4 is also reflected in the fact that it can be used as a biomarker of poor prognosis in triple‐negative breast cancer together with LXRβ.^33^ It is particularly noteworthy that the function of CLDN4 varies with different tissue environments, and it can even inhibit tumors in some cancers. For example, low CLDN4 expression in gastric cancer is associated with poor tumor differentiation and increased metastasis,^29^ whereas in pancreatic cancer, it has been shown to inhibit invasion and metastasis.^30^ These different effects indicate that the biological function of CLDN4 depends on the specific tissue microenvironmental network. The imbalance of CLDN4 expression found in our research makes it a potential therapeutic target. Gain‐ and loss‐of‐function experiments confirmed that CLDN4 is a key driver of malignant phenotypes in PTC. Overexpression of CLDN4 enhances cell proliferation and migration and inhibits cell apoptosis, which are related to tumor progression and treatment resistance, further supporting its therapeutic potential.

Importantly, miR‐941 was identified as a direct upstream regulator of CLDN4, which targets the 3′‐UTR of CLDN4 mRNA to suppress its expression and consequently reverses CLDN4‐driven malignancy. MiR‐941 is known as a uniquely human miRNA that has been implicated in evolution and the regulation of neurotransmitter signaling,^16^ however, its role in cancer remains poorly understood. Comparative analysis shows that miR‐941 expression in tumor tissues is about eight‐fold higher than in adjacent normal tissue, accompanied by the inhibition of several tumor suppressor genes.^31^ In gastric cancer, the down‐regulation of miR‐941 may inhibit malignant tumors by targeting KDM6B and TAOK1.^34^ In contrast, the expression pattern of miR‐941 in hepatocellular carcinoma remains controversial, with some studies reporting its downregulation,^17^ and others demonstrating its upregulation.^35^ Our study extended the function of miR‐941 to thyroid cancer and identified CLDN4 as a key downstream target. This explains why CLDN4 is overexpressed, probably because miR‐941 is silenced or down‐regulated. These findings suggest that therapeutic strategies such as miRNA mimics or demethylating agents, which restore miR‐941 expression and subsequently target CLDN4, may hold promise for the treatment of thyroid cancer.

Here, a functional relationship is described between the oncogenic activity of CLDN4 and the PI3K/AKT pathway, which is frequently over‐activated in thyroid cancer. Abnormal activation of the PI3K/AKT signal is a recognized driving factor of thyroid tumors, which is usually caused by mechanisms such as PIK3CA mutation, PTEN deletion or inactivation, and excessive growth factor receptor signals.^24^, ^36^ This pathway plays a key role in maintaining and developing thyroid tumors by regulating cell proliferation, survival, metabolic adaptation, and protein synthesis.^37^ Based on the observed dysregulation of CLDN4, its role in promoting PI3K/AKT activation was further elucidated. Overexpression of CLDN4 increased the levels of phosphorylated PI3K and AKT, and inhibited the expression of PTEN in cell and mouse ectopic tumor models. Because PTEN is the main negative regulator of this pathway, its down‐regulation seems to be very important to maintain the activation of PI3K/AKT. Importantly, the CLDN4‐driven oncogene phenotype was effectively reversed by PI3K inhibitor LY294002 in vitro and in vivo, which confirmed its functional dependence on the PI3K/AKT signal. In addition, CLDN4‐induced PI3K/AKT activation was found to be blocked by miR‐941. Notably, the M2‐skewing phenotype we observed upon CLDN4 overexpression is consistent with the established role of PI3K/AKT/mTOR signaling in promoting alternative (M2) macrophage polarization, whereas AKT inhibition favors the M1 phenotype. Our co‐culture findings therefore position CLDN4‐driven PI3K/AKT activation as a tumor‐cell‐intrinsic mechanism that shapes the tumor immune microenvironment, potentially contributing to immune evasion in PTC. In a word, these results reveal a consistent regulatory axis from miR‐941 to CLDN4 and finally to the PI3K/AKT signal, which provides a new conceptual framework for us to understand the molecular network of thyroid cancer.

Our findings strongly support the evaluation of these innovative treatments, especially for advanced or refractory thyroid cancer. It should be acknowledged that this study has some limitations. First, the precise molecular mechanism by which CLDN4 activates the PI3K/AKT pathway remains to be elucidated. As CLDN4 lacks intrinsic enzymatic activity, it may indirectly downregulate PTEN by modulating E3 ubiquitin ligases such as NEDD4‐1, WWP1, or XIAP, which promote PTEN polyubiquitination and subsequent degradation. Given that multiple E3 ligases and deubiquitinating enzymes (DUBs) coordinately regulate PTEN ubiquitination status and consequent AKT activity,^38^, ^39^ it is plausible that CLDN4 is involved in this regulatory network. However, the precise mechanisms through which CLDN4 engages these ubiquitination modifiers in thyroid cancer warrant further investigation.

Second, as the investigation was primarily focused on the PI3K/AKT pathway, CLDN4 may also regulate additional mechanisms that require further exploration. As a barrier‐forming Claudin protein, CLDN4 can inhibit cation channels formed by CLDN2 or CLDN15. Disruption of polymeric complexes comprising CLDN2, CLDN7, CLDN15, and CLDN19 may compromise barrier integrity, thereby reducing paracellular permeability and potentially impeding drug penetration into the tumor microenvironment.^40^ Meanwhile, CLDN4 is a significant contributor to immune evasion within the tumor microenvironment. In ovarian cancer, it dampens anti‐tumor immune signaling and reduces CD8+ T cell infiltration by modulating the Rab7/STING/type I interferon pathway.^41^ Furthermore, CLDN4 overexpression impairs T cell function through inhibition of the T cell receptor zeta‐chain, while therapeutic targeting of CLDN4 restores this pathway, promotes CD8+ T cell infiltration, and synergizes with PARP inhibitors such as niraparib to exert robust anti‐tumor efficacy.^42^ Additionally, while our animal models enabled assessment of primary tumor growth, future research should develop metastatic models to more comprehensively evaluate CLDN4's role in distant metastasis.

Third, the malignant identity of tumor‐derived epithelial cells was inferred from transcriptional profiles and sample origin rather than formally validated by CNV‐based methods such as inferCNV or CopyKAT. Future studies should incorporate CNV inference to more rigorously distinguish malignant from non‐malignant epithelial cells within the tumor microenvironment.

Fourth, the PI3K/AKT pathway was inhibited only by LY294002 in this study. Although LY294002 is a widely used PI3K‐specific inhibitor, future studies using additional pharmacological inhibitors (e.g., wortmannin) are needed to further confirm the specificity of the pathway conclusions.

In general, this study demonstrates that CLDN4 functions as a significant oncogenic driver in thyroid cancer. Its expression is negatively regulated by miR‐941, and it promotes tumor progression through activation of the PI3K/AKT signaling pathway. These findings not only enhance our understanding of thyroid carcinogenesis but also provide a foundation for developing novel diagnostic and therapeutic strategies targeting CLDN4.

## 5 CONCLUSIONS

In summary, this study integrates single‐cell and bulk transcriptomic analyses with functional validation to reveal a CLDN4‐high malignant epithelial subpopulation associated with PI3K/AKT pathway activation in PTC. We demonstrate that miR‐941 directly targets CLDN4 to suppress tumor progression both in vitro and in vivo, and that CLDN4 activates the PI3K/AKT signaling pathway to promote malignant phenotypes. Clinical validation in 25 paired PTC tissues confirmed that CLDN4 is significantly upregulated while miR‐941 is downregulated, with high CLDN4 expression correlating with larger tumor size, lymphatic metastasis, and vascular invasion. Furthermore, we provide evidence that CLDN4 expression in tumor cells promotes M2 macrophage polarization while suppressing M1 polarization, suggesting a role in remodeling the tumor immune microenvironment. Collectively, these findings establish the miR‐941/CLDN4/PI3K/AKT axis as a critical regulator of PTC progression and highlight CLDN4 as a promising therapeutic target for precision oncology.

## AUTHOR CONTRIBUTIONS

**Jinghua Zhou**: Formal analysis; investigation; writing—original draft. **Tiancheng Fu**: Formal Analysis; investigation; writing—original draft; writing—review and editing. **Guoxiang Huang**: Investigation; methodology; writing—review and editing. **Guangfeng Luo**: Formal Analysis; writing—review and editing. **Chengcheng Wang**: Data curation; writing—review and editing. **Yanze Wang**: Formal Analysis; investigation; writing—review and editing. **Ziyang Ding**: Formal Analysis; Investigation; Writing—review and editing. **Xiarong Hu**: Conceptualization; supervision; writing—review and editing. **Yudong Li**: Conceptualization; project administration; resources; supervision; writing—review and editing. **Zhen Li**: Conceptualization; project administration; resources; supervision; writing—review and editing.

## CONFLICT OF INTEREST STATEMENT

The authors declare no conflicts of interest.

## ETHICS STATEMENT

All subjects gave their informed consent for inclusion before they participated in the study. The study was conducted in accordance with the Declaration of Helsinki, and the research protocol has been approved by the Research Ethics Committee of the Tenth Affiliated Hospital of Southern Medical University (KYKT2025‐110). Prior to study commencement, informed consent was obtained in writing from each participant.

## Supporting information

Supporting Information S1

## Data Availability

The data that support the findings of this study are available from the corresponding author upon reasonable request.
