# Supplementary material for: Integrated single‐cell transcriptomics reveals miR‐941 targets CLDN4 to inhibit malignant progression of thyroid cancer through the PI3K/AKT signaling pathway
Source: J Cell Commun Signal. 2026 Oct 27;20(4):e70123. doi: 10.1002/ccs3.70123 (PMC13714453; doi:10.1002/ccs3.70123)
Supplement: Supplementary file 1 [file CCS3-20-e70123-s001.docx]

qPCR and WB were used to detect the mRNA and protein expression levels of CLDN4 after shRNA treatment.
